# Immune Therapeutic Strategies in Chronic Hepatitis B Virus Infection: Virus or Inflammation Control?

**DOI:** 10.1371/journal.ppat.1003784

**Published:** 2013-12-19

**Authors:** Antonio Bertoletti, Adam J. Gehring

**Affiliations:** 1 Program in Emerging Viral Diseases, Duke-NUS Graduate Medical School, Singapore; 2 Viral Hepatitis Unit, Singapore Institute for Clinical Sciences, Agency for Science, Technology and Research (A*Star), Singapore; 3 Molecular Microbiology and Immunology Department, Saint Louis University School of Medicine, St. Louis, Missouri, United States of America; Duke University Medical Center, United States of America

More than 240 million people worldwide are infected with hepatitis B virus (HBV) and are at risk of developing liver cirrhosis and hepatocellular carcinoma (HCC). Reducing this pool of infected people is a necessity since despite an effective prophylactic vaccine, about 2% of the vaccinated individuals in high endemic areas still develop chronic HBV infection (CHB). A battery of antiviral drugs based on nucleoside or nucleotide analogues that target the HBV reverse transcriptase are available. They efficiently suppress HBV replication and reduce liver inflammation linked with cirrhosis but rarely achieve virus eradication. HBV is present in hepatocytes in a mini-chromosomal form (called cccDNA) that is untouched by reverse transcriptase inhibitors. As a consequence, response to the treatment is hardly durable and a majority of patients experience HBV reactivation when antiviral therapy is withdrawn [1]. Failure to achieve sustained control of HBV infection is linked with an inability to elicit an effective immune response that resembles one present in adult patients that resolve acute infection. However, since HBV is a non-cytopathic virus, immunological processes are also responsible for the chronic inflammatory events that cause cirrhosis and HCC [1]. Immunotherapeutic approaches primarily designed to control viral replication through the boosting of antiviral immunity or that aim to inhibit the liver inflammatory processes linked with cirrhosis and HCC development will be discussed.

## What Is the Immunological Profile of HBV Control?

Efficient control of HBV infection is associated with the induction and persistence of helper and cytotoxic T cells targeting different HBV proteins and production of anti-HBV envelope antibodies. This antiviral immune response is composed of T cells able to secrete Th1 cytokines, proliferate and lyse HBV infected hepatocytes [1], and it is induced almost exclusively in adult patients after acute HBV infection. Chronic HBV patients fail to mount such an efficient antiviral response. HLA-class II genetic profile [2], dose of virus [3], and age at infection [4] influence the induction of a protective antiviral immunity, but a detailed discussion of the causes of HBV chronicity exceeds the focus of this brief review. What has instead been clearly demonstrated in animal models and in natural infection is that HBV-specific adaptive immunity occurs in the context of a peculiar innate immune response. This response is delayed four to six weeks post infection when HBV replication has already reached extremely high levels (>10^6^ copies/ml) [1]. Innate immune activation is characterized by large production of IFN-γ rather than IFN-α/β [5]. Indeed, while HBV doesn't trigger high IFN-α production and also interferes with its antiviral effect [6], [7], a robust IFN-γ production precedes the detection of HBV-specific T cells in acute HBV and correlates with a significant drop in HBV replication and HBV antigens [5]. The innate immune cell components responsible for HBV sensing and IFN-γ production and ultimately induction of protective adaptive immunity have not been defined during natural infection [1]. Elegant work in mice showed that CD1-restricted NKT cells are activated by self-lipids induced by HBV replication [8], but in addition to the fact that HBV replication in this mouse model was mediated by an adenoviral vector, CD1-restricted NKT cells are abundant in mouse but not in human livers [9]. In human, the network of innate lymphocytes resident in the liver is mainly composed of NK CD56 bright cells and mucosal-associated invariant T (MAIT) cells that can produce large quantities of IFN-γ through cytokine-mediated (IL-12, IL-18) activation [10]. Activation of these innate cells might therefore be necessary for the successful control of HBV infection, but further studies are necessary to precisely define their role in the early stage of infection.

## What Can Be the Strategies to Restore Adaptive Immunity in Chronic HBV Patients?

**Figure 1:**
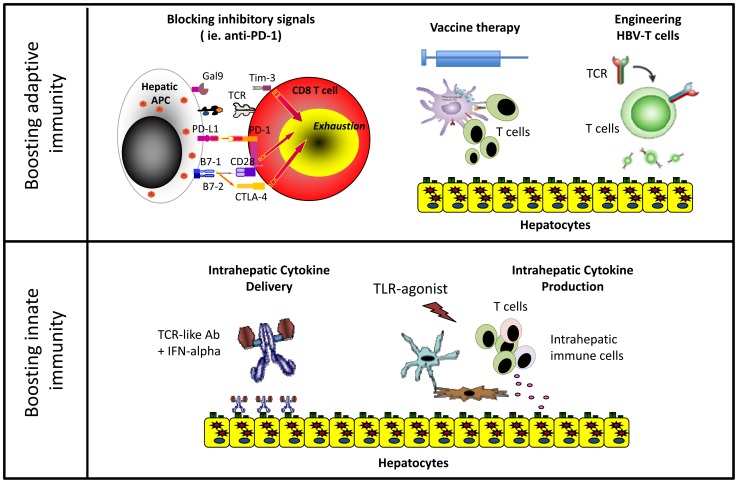
Different immune-based therapeutic strategies aiming to increase HBV control.

After years of exposure to HBV antigens, HBV-specific T cells are deleted or functionally exhausted in chronic patients. Virus-specific T cells express inhibitory molecules like PD-1 [11], CTLA-4 [12], SLAM [13], and TIM-3 [14], [15] and are defective in proliferation and cytokine production. Blocking inhibitory pathways, new aggressive vaccination regimens and experimental gene therapy strategies have been proposed to rebuild a functional HBV-specific T cell response in these patients (Figure 1).

Blocking inhibitory pathways associated with T cell exhaustion has shown therapeutic efficacy in cancer patients [16]. Interfering with these pathways can also achieve partial functional recovery of HBV-specific T cells from CHB patients in vitro, but we still lack data in vivo evaluating the efficacy of this approach in CHB patients [16].

**Figure 2:**
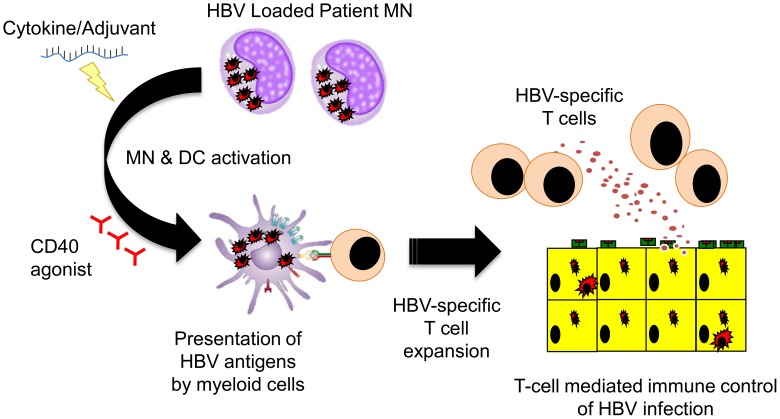
Schematic representation of the potential ability of HBV-loaded monocytes [22] to stimulate HBV-specific T cells trough TLR [20] or anti-CD40 agonists [21]. MN = monocytes, DC = dendritic cells.

Vaccine therapy aims to induce functionally efficient HBV-specific T cells on the background of virus-specific T cell exhaustion. Several strategies have been tested in clinical trials with disappointing results. Often, vaccine therapy did not induce an HBV-specific T cell response, or when such response was boosted, it did not have a therapeutic effect [17]. However, it was recently demonstrated that the use of new highly immunogenic vaccine preparations in combination with antiviral treatment showed immunological and therapeutic efficacy in the woodchuck model of chronic HBV infection [18]. In addition, intriguing data was reported from a phase III clinical trial of a therapeutic vaccine. A significant virological response was observed in 20% of patients treated not only with the vaccine but also with adjuvant alone [19]. The immunological mechanisms responsible were not characterized but plausible hypotheses can be derived from recent studies. Intrahepatic activation of the myeloid compartment with TLR agonists [20] results in effective T cell expansion in murine systems. Agonistic anti-CD40 [21] activation of dendritic cells rescues naïve CD8 T cell priming to antigens produced in the liver of HBV transgenic mice, which is otherwise aborted by PD-1/PD-L1 interactions. These data together with our recent demonstration that monocytes internalize HBV antigens in the circulation of chronic patients and activate autologous HBV-specific T cells after maturation with inflammatory stimuli [22] suggest that repetitive injections of adjuvants alone could induce the inflammatory environment capable of stimulating intrahepatic HBV-specific T cells (Figure 2). This could provide a clear advantage to current vaccines that use a single recombinant antigen and do not account for the 8% of genetic diversity among HBV genotypes [23]. Capitalizing on the antigen present in infected patients could provide the full repertoire of antigens personalized to the infecting virus. If this can be confirmed in vivo, CHB vaccine therapy could be performed using adjuvants alone and the “personalized antigenic depot” within the patients to overcome the problem of viral diversity.

However, more radical approaches could be needed to circumvent HBV-specific T cell deletion in patients with high viral loads. Engineering HBV-specific T cells through transfer of HBV-specific T cell receptors or HBV-specific chimeric antigen receptors (CAR) showed encouraging results in vitro and in animal models [24], [25]. The concept of adoptively transferring a functionally efficient HBV-specific immune system is not new in HBV. CHB patients receiving bone marrow transplants from HBV-immune donors were cured. Likewise, HBV+ livers transplanted in HBV-immune donors resulted in viral control [17]. Thus, gene therapy approaches might have potential, but safety concerns, cost, and ethical issues related to viral vector use need to be addressed.

Several other factors have also hampered the restoration of efficient adaptive immunity in CHB. Epigenetic programs were suggested to preserve the T cell exhaustion state in chronic viral infection [26]. Also, in many CHB patients, HBV-specific T cells are not only dysfunctional but can be physically deleted, adding obstacles to vaccination approaches. Furthermore, IL-10 [16], TGF-β [27], arginase [28], and T regulatory cells [29] are increased in the chronically inflamed livers. Even if it is possible to genetically engineer HBV-specific immunity, the suppressive microenvironment could hinder HBV clearance.

The duration of exposure and antigen burden in chronic HVB infection are believed to be the primary factors affecting virus-specific T cell function. Thus, modulating the quantity of viral antigens secreted by HBV might have a beneficial effect as has been observed in HBV transgenic mice [30]. It should also be considered that adolescent and young CHB patients display a less compromised HBV-specific antiviral immune response than their adult counterparts [31]; therefore, they may be more responsive to immunotherapeutic strategies.

## How Can We Directly Stimulate Intrahepatic Innate Immunity?

The important role played by innate immunity in the early stages of infection has stimulated therapeutic strategies to specifically target this branch. IFN-α therapeutic efficacy is associated with NK cell activation [32]. Therefore, increasing intrahepatic IFN-α levels could be clinically beneficial (Figure 1). In one approach, we have developed T cell receptor–like antibodies conjugated with IFN-α that specifically target HBV-infected hepatocytes to increase intrahepatic IFN-α delivery [33].

Other approaches in clinical development seek to induce intrahepatic IFN-α production through oral administration of TLR agonists. TLR7 agonists stimulate robust IFN-α production in plasmacytoid dendritic cells. However, prolonged efficacy in TLR-7 agonist–treated, HBV-infected chimpanzees was not only related to the production of IFN-α, as its production was transient and not liver specific. Antiviral efficacy was more consistent with a boost of intrahepatic NK, NKT, and T cell responses associated with production of IFN-γ [34]. Since, as reported previously, intrahepatic NK and MAIT cells can be activated by IL-12 and IL-18 cytokines [10], TLR agonists triggering IL-12 and IL-18 hepatic secretion might be particularly important. These cytokines selectively activate innate immune cells within the liver compartment and can induce partial functional recovery of exhausted HBV-specific CD8^+^ T cells [35].

## Is the Reduction of Liver Inflammation a Valuable Therapeutic Strategy for CHB Infection?

The immunotherapeutic strategies we reviewed aim to control HBV infection by increasing antiviral immunity and as such terminate the chronic inflammatory process. However, activation of intrahepatic adaptive or innate cellular immunity requires tight control because it could also exacerbate liver inflammation. Even though mechanisms like IL-10 production, release of arginase from hepatocytes, and even the dampening of T cell responses by activated NK cells [36] are in place to control excessive activation of intrahepatic immunity, hepatocyte lysis and increased IFN-γ production can still trigger inflammatory chemokines (e.g., CXCL10) responsible for recruiting inflammatory cells (macrophages, non-HBV-specific T cells) that cause the bulk of liver damage [1].

A radically different perspective in chronic HBV treatment is to consider CHB a necro-inflammatory rather than viral disease. Recent data in HBV transgenic mice clearly indicate that suppressing intrahepatic CTL activity in the liver using anti-platelet therapy prevents hepatocellular carcinoma. Platelets promote the accumulation of CD8 T cells in the liver and anti-platelet therapy blocks this process, reducing hepatocellular injury and fibrosis [37]. In addition to these experimental data, observations that HBsAg quantity is associated with liver fibrosis protection [38] introduced a further level of uncertainty about the beneficial effect of HBV suppression.

In conclusion, the current immunotherapeutic strategies designed to suppress and control HBV replication have strong scientific support. However, they are restricted by our limited knowledge, and further understanding of the relationship with the virus in the unique environment of the human liver will almost certainly provide opportunities to enhance them or perhaps develop totally novel approaches.
